# Supplementary material for: Deep convolutional neural networks for accurate somatic mutation detection
Source: Nat Commun. 2019 Mar 4;10:1041. doi: 10.1038/s41467-019-09027-x (PMC6399298; doi:10.1038/s41467-019-09027-x)
Supplement: Supplementary file 1 — Supplementary Information [file 41467_2019_9027_MOESM1_ESM.pdf]

Sahraeian et al. Deep convolutional neural networks for accurate somatic mutation detection.

SUPPLEMENTARY INFORMATION

Supplementary Figures

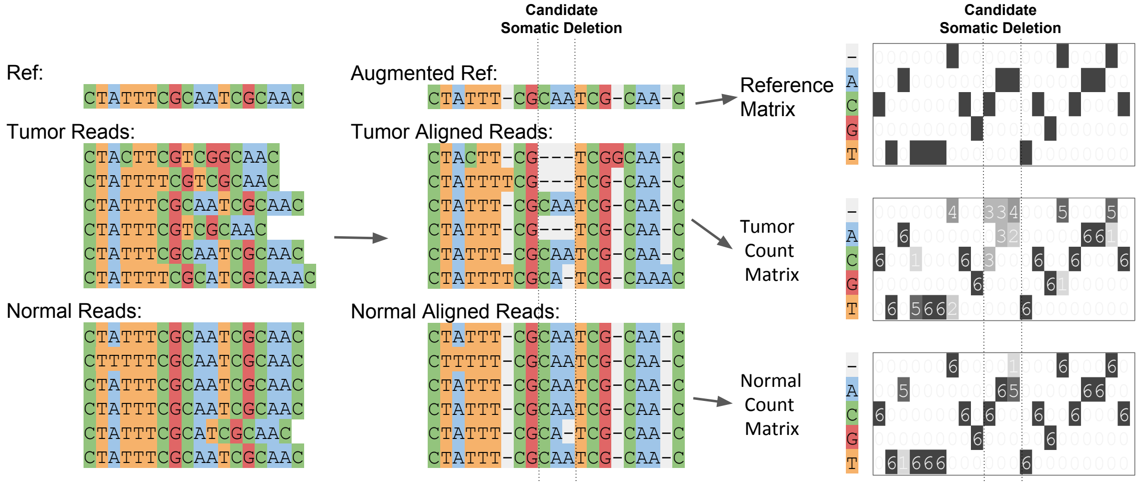

Supplementary Figure 1: Toy example of input matrix preparation for a given candidate somatic deletion. Sequence alignment information in a window of 7 bases around the candidate somatic mutation is extracted. The reference sequence is then augmented by adding gaps to account for insertions in the reads. The augmented alignment is then summarized into the reference matrix, the tumor count matrix, and the normal count matrix. The count matrices record the number of A/C/G/T and gap ('-') characters in each column of the alignment, while the reference matrix records the reference bases in each column. The count matrices are then normalized by coverage to reflect base frequencies in each column. Separate channels are reserved to record the tumor and normal coverages.

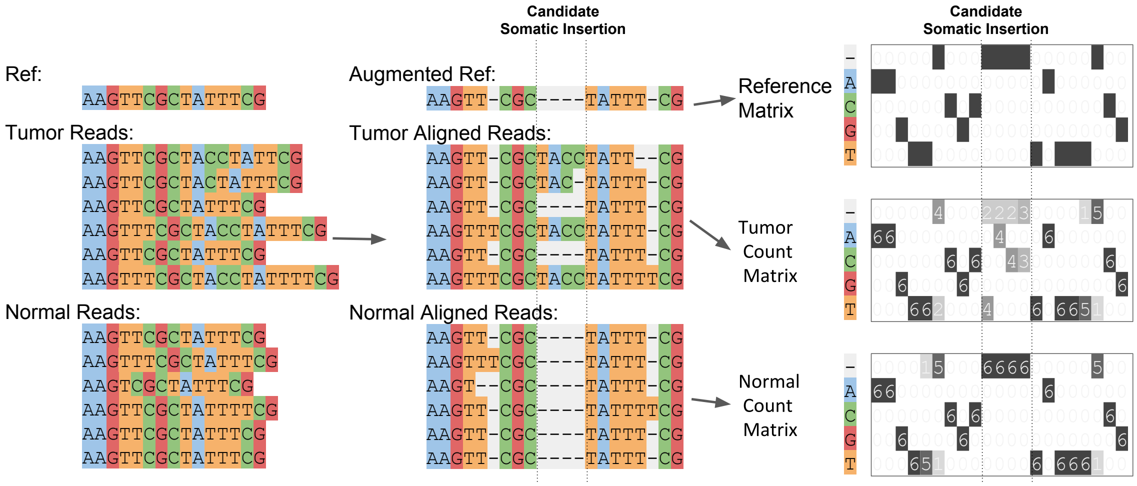

Supplementary Figure 2: Toy example of input matrix preparation for a given candidate somatic insertion. Sequence alignment information in a window of 7 bases around the candidate somatic mutation is extracted. The reference sequence is then augmented by adding gaps to account for insertions in the reads. The augmented alignment is then summarized into the reference matrix, the tumor count matrix, and the normal count matrix. The count matrices record the number of A/C/G/T and gap ('-') characters in each column of the alignment, while the reference matrix records the reference bases in each column. The count matrices are then normalized by coverage to reflect base frequencies in each column. Separate channels are reserved to record the tumor and normal coverages.

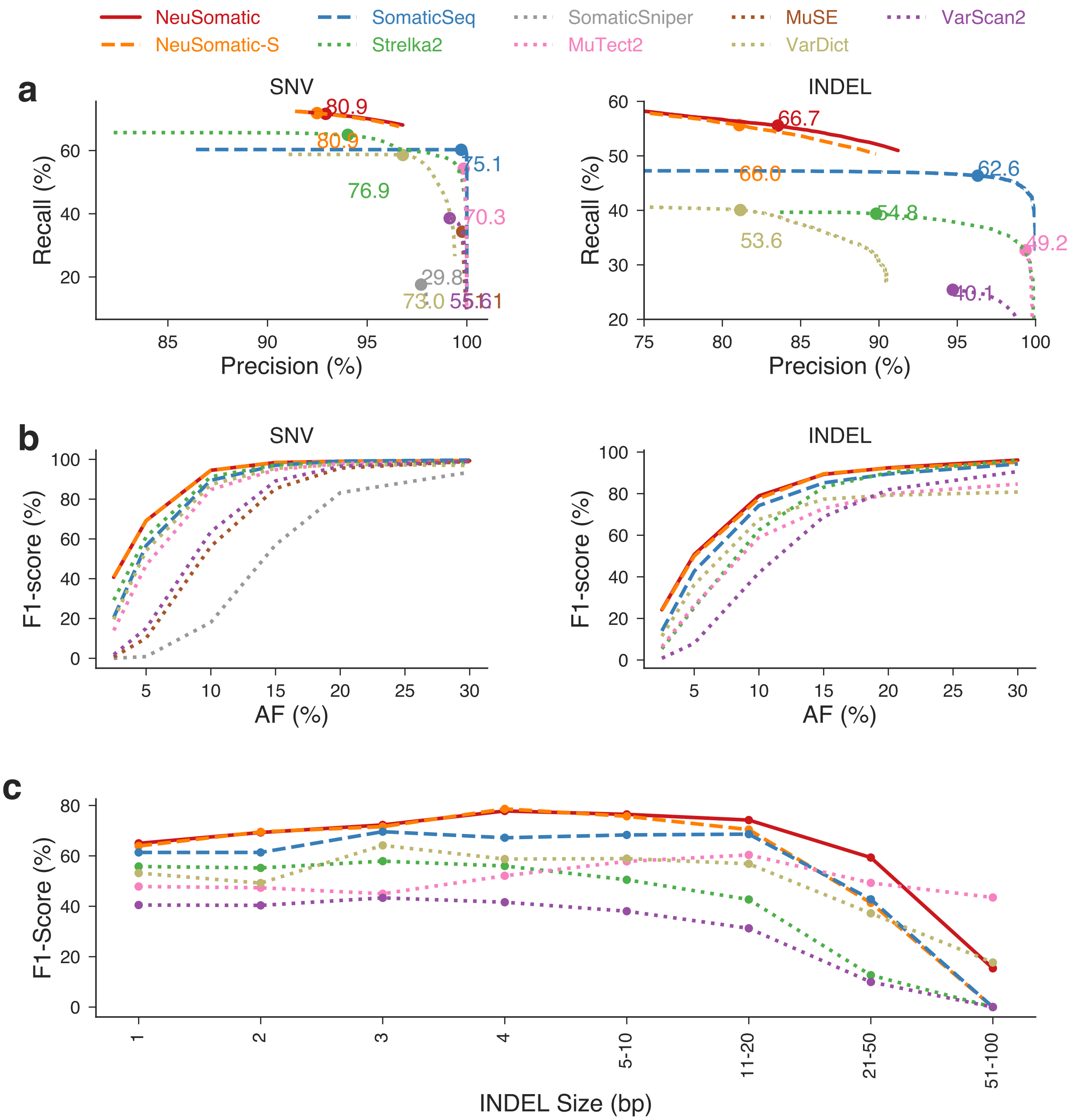

Supplementary Figure 3: Performance analysis of the Platinum tumor spike dataset. In this dataset, reads are spiked with frequencies sampled from a binomial distribution with means $[0.05, 0.1, 0.2, 0.3]$, while normal sample is pure. (a) Precision-recall analysis: the confidence or quality scores are used to derive the precision-recall curves. The highest F1-score achieved by each algorithm is printed on the curve and marked with a solid circle. (b) Performance analysis for different AFs. (c) Performance analysis of INDEL accuracy (F1-score) for different INDEL sizes.

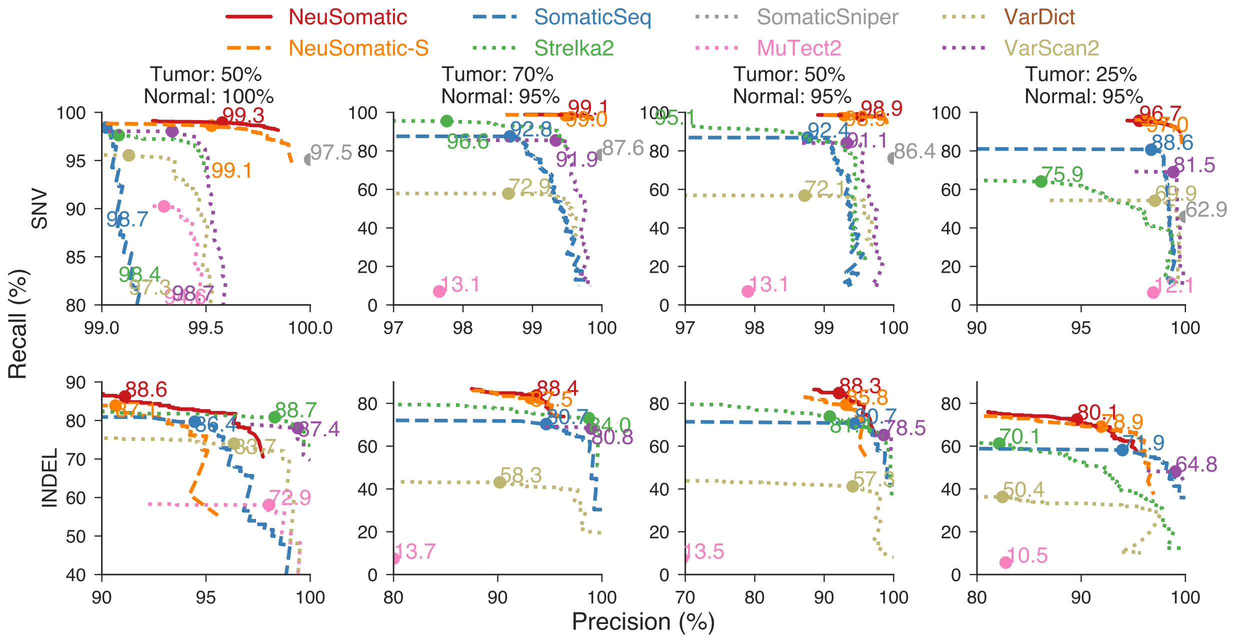

Supplementary Figure 4: Performance analysis of exome sample mixture. In this dataset, four tumor and normal purity scenarios (50%T:100%N, 70%T:95%N, 50%T:95%N and 25%T:95%N) are used. The confidence or quality scores are used to derive the precision-recall curves. The highest F1-score achieved by each algorithm is printed on the curve and marked with a solid circle. Here the training is on exome data for NeuSomatic, NeuSomatic-S, and SomaticSeq.

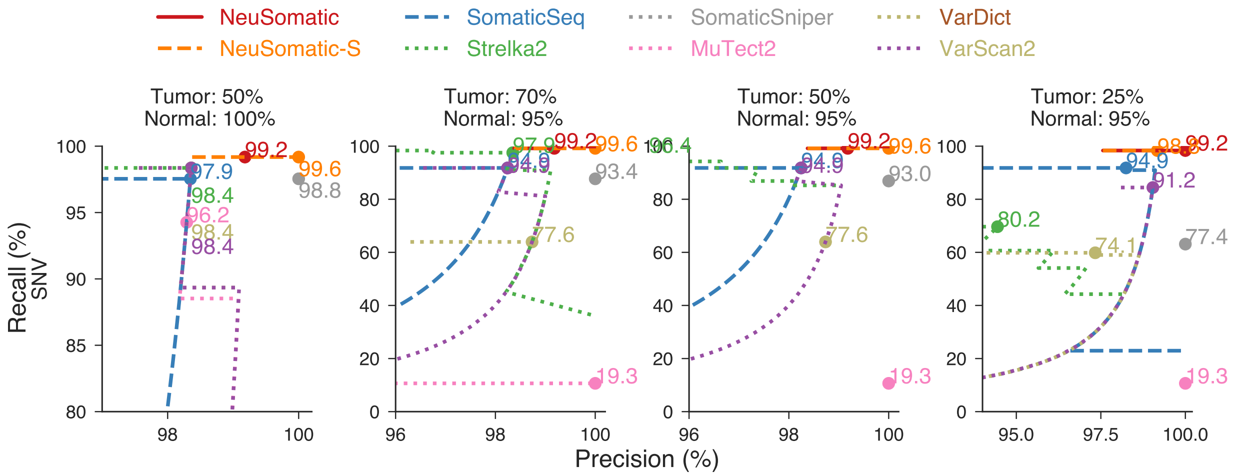

Supplementary Figure 5: Performance analysis of Target panel sample mixture. In this dataset, four tumor and normal purity scenarios (50%T:100%N, 70%T:95%N, 50%T:95%N and 25%T:95%N) are used. The confidence or quality scores are used to derive the precision-recall curves. The highest F1-score achieved by each algorithm is printed on the curve and marked with a solid circle. Here the training is on exome data for NeuSomatic, NeuSomatic-S, and SomaticSeq.

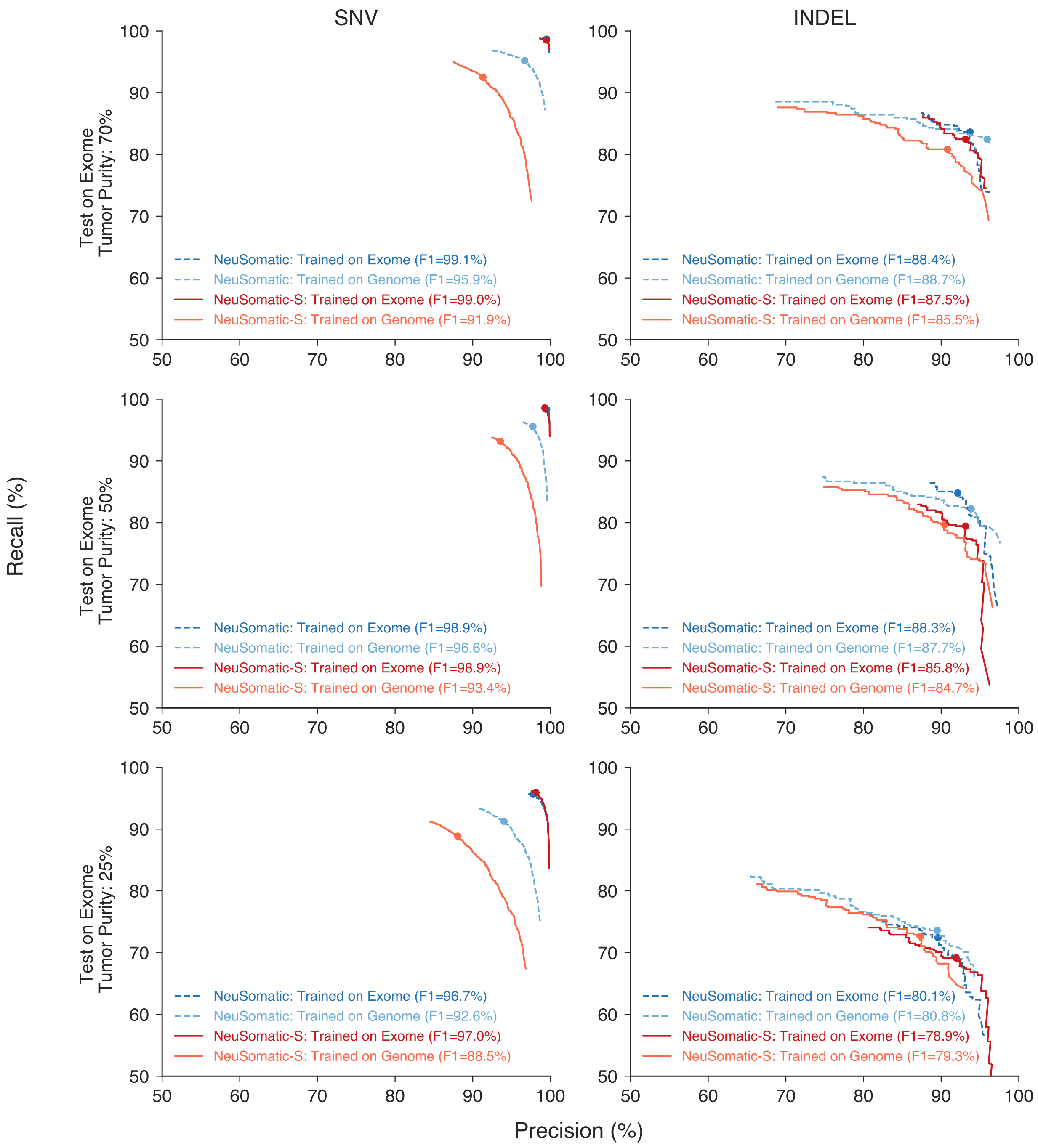

Supplementary Figure 6: Performance analysis of using models trained on whole-genome (Platinum data, genome mixture) and whole-exome (HG003-HG004 exome mixture) to test on exome mixture dataset. The confidence or quality scores are used to derive the precision-recall curves. The highest F1-score achieved by each algorithm is indicated in the legend and marked with a solid circle on the curve.

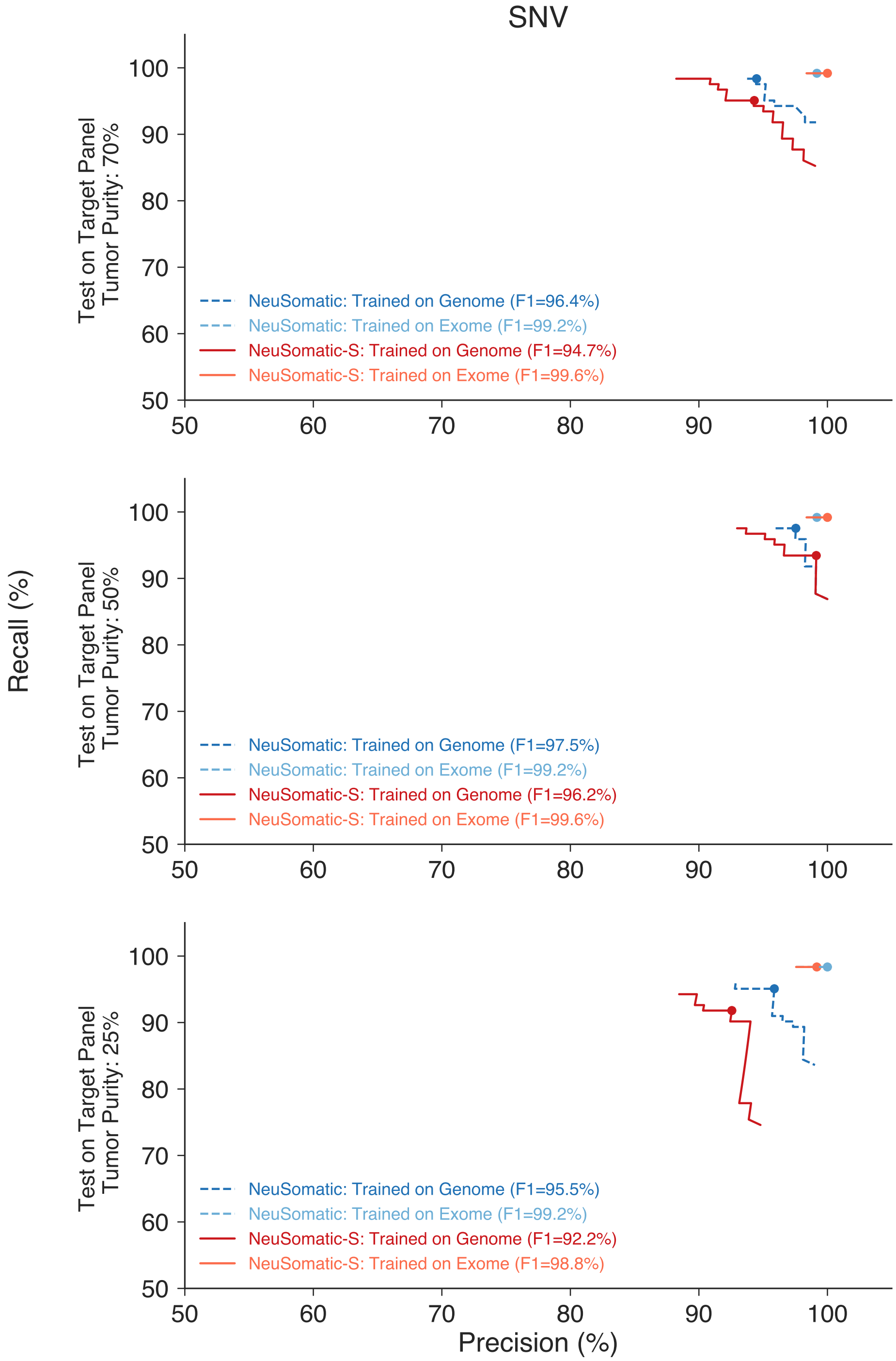

Supplementary Figure 7: Performance analysis of using models trained on whole-genome (Platinum data, genome mixture) and whole-exome (HG003-HG004 exome mixture) to test on target panel mixture dataset. The confidence or quality scores are used to derive the precision-recall curves. The highest F1-score achieved by each algorithm is indicated in the legend and marked with a solid circle on the curve.

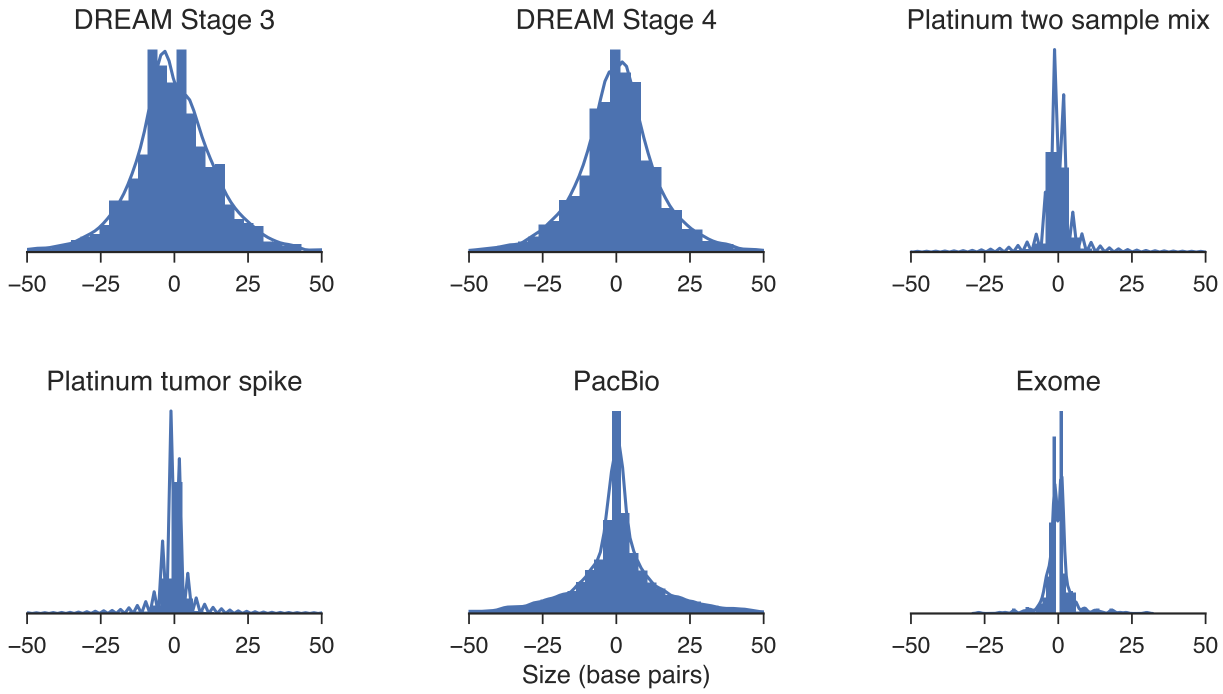

Supplementary Figure 8: Size distribution of ground truth INDELs in Dream Stage 3, Dream Stage 4, Platinum two sample mixture, Platinum tumor spike, PacBio, and exome datasets. Negative sizes corresponds to deletions.

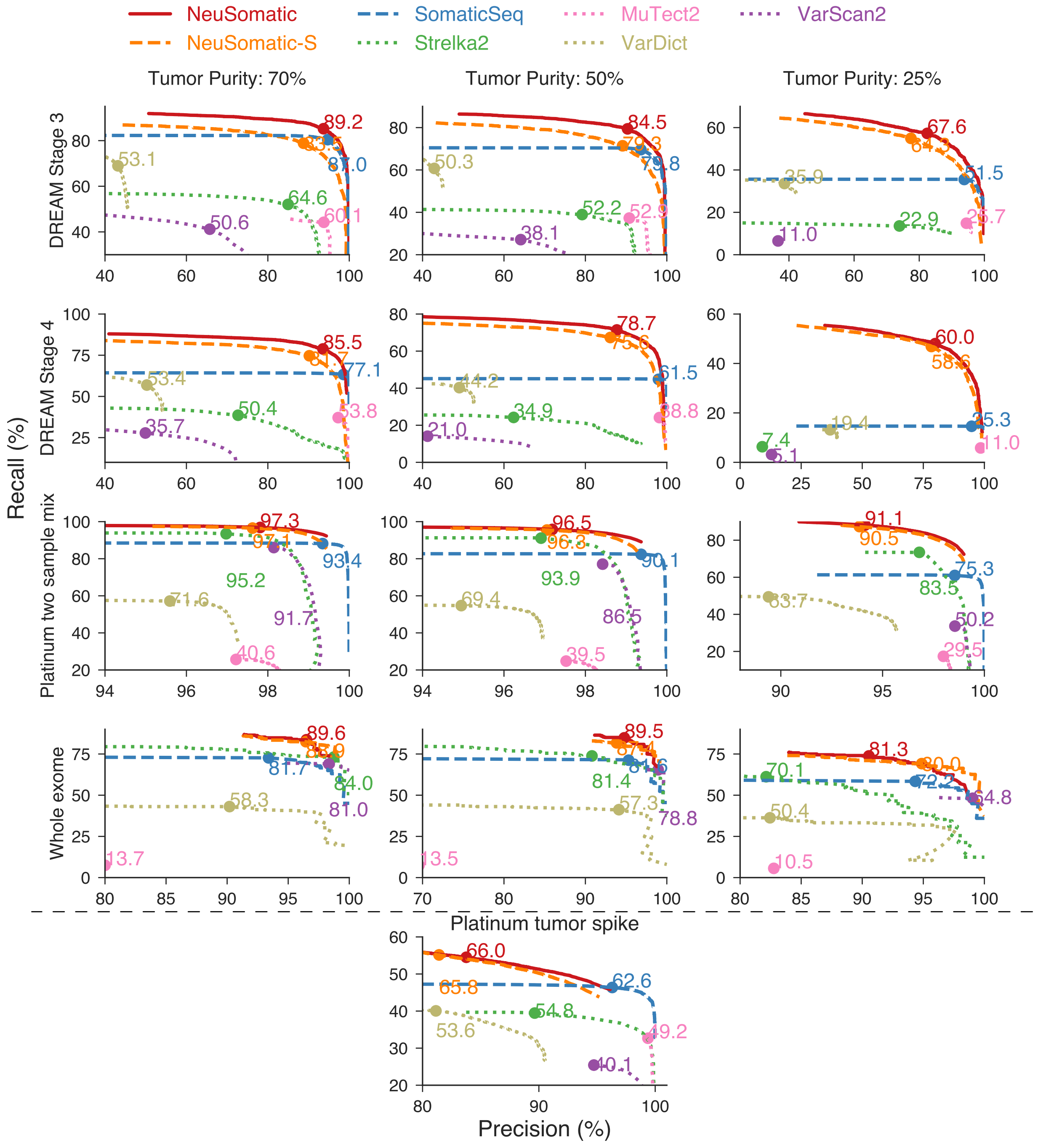

Supplementary Figure 9: Performance analysis of INDELs based on position and type of the predicted somatic mutations (while ignoring the accuracy of the exact predicted INDEL sequence) for Dream Stage 3, Dream Stage 4, Platinum two sample mixture, whole-exome, and Platinum tumor spike datasets. For the first four datasets, three tumor purity scenarios (70%, 50% and 25%) are used while normal sample has 95% purity. The confidence or quality scores are used to derive the precision-recall curves. The highest F1- score achieved by each algorithm is printed on the curve and marked with a solid circle.

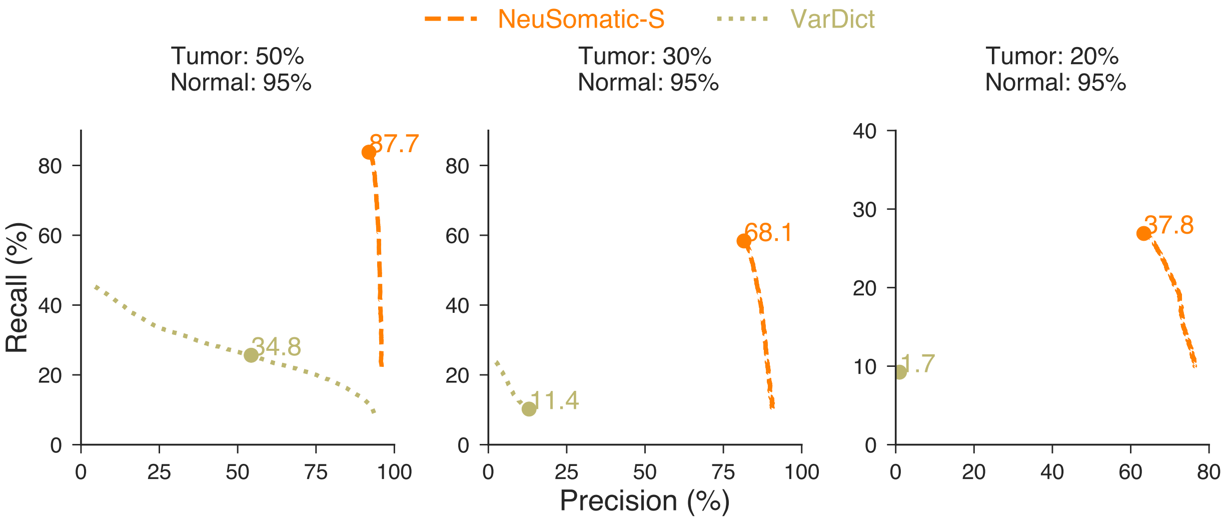

Supplementary Figure 10: Performance analysis of INDELs based on position and type of the predicted somatic mutations (while ignoring the accuracy of the exact predicted INDEL sequence) for PacBio dataset on three tumor purity scenarios (50%, 30% and 20%) and 95% normal purity. The confidence or quality scores are used to derive the precision-recall curves. The highest F1- score achieved by each algorithm is printed on the curve and marked with a solid circle.

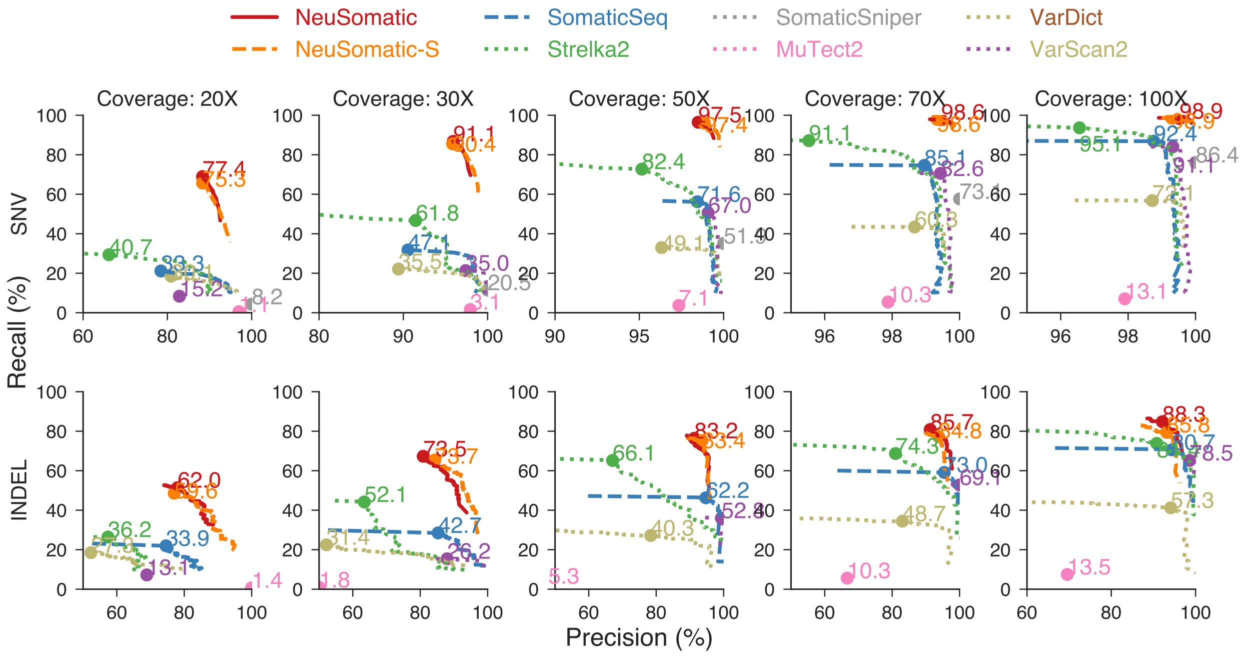

Supplementary Figure 11: Performance analysis of the sequence coverage impact on the whole-exome sample mixture dataset. In this example, tumor has 50% purity and normal has 95% purity. Tumor and normal alignments coverages are ranging from 20× to 100×. The confidence or quality scores are used to derive the precision-recall curves. The highest F1-score achieved by each algorithm is indicated in the legend and marked with a solid circle on the curve.

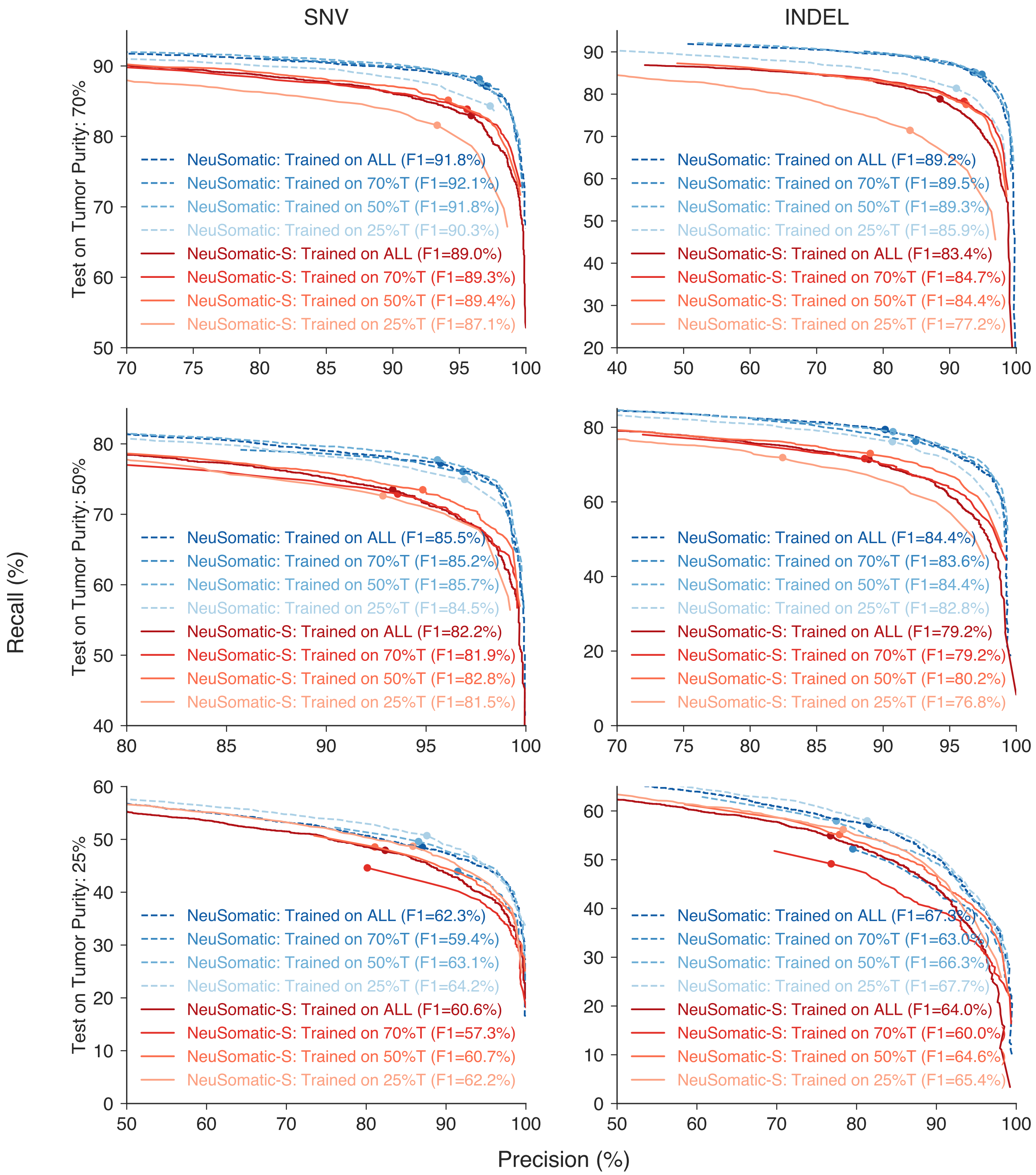

Supplementary Figure 12: Performance analysis of cross sample training for Dream challenge Stage 3 dataset. We tested each of the samples with tumor purities of 70%, 50%, and 25% with NeuSomatic models trained on different purities, as well as a model trained on collective inputs from all different purities. The confidence or quality scores are used to derive the precision-recall curves. The highest F1-score achieved by each algorithm is printed in the legend and marked with a solid circle on the curve.

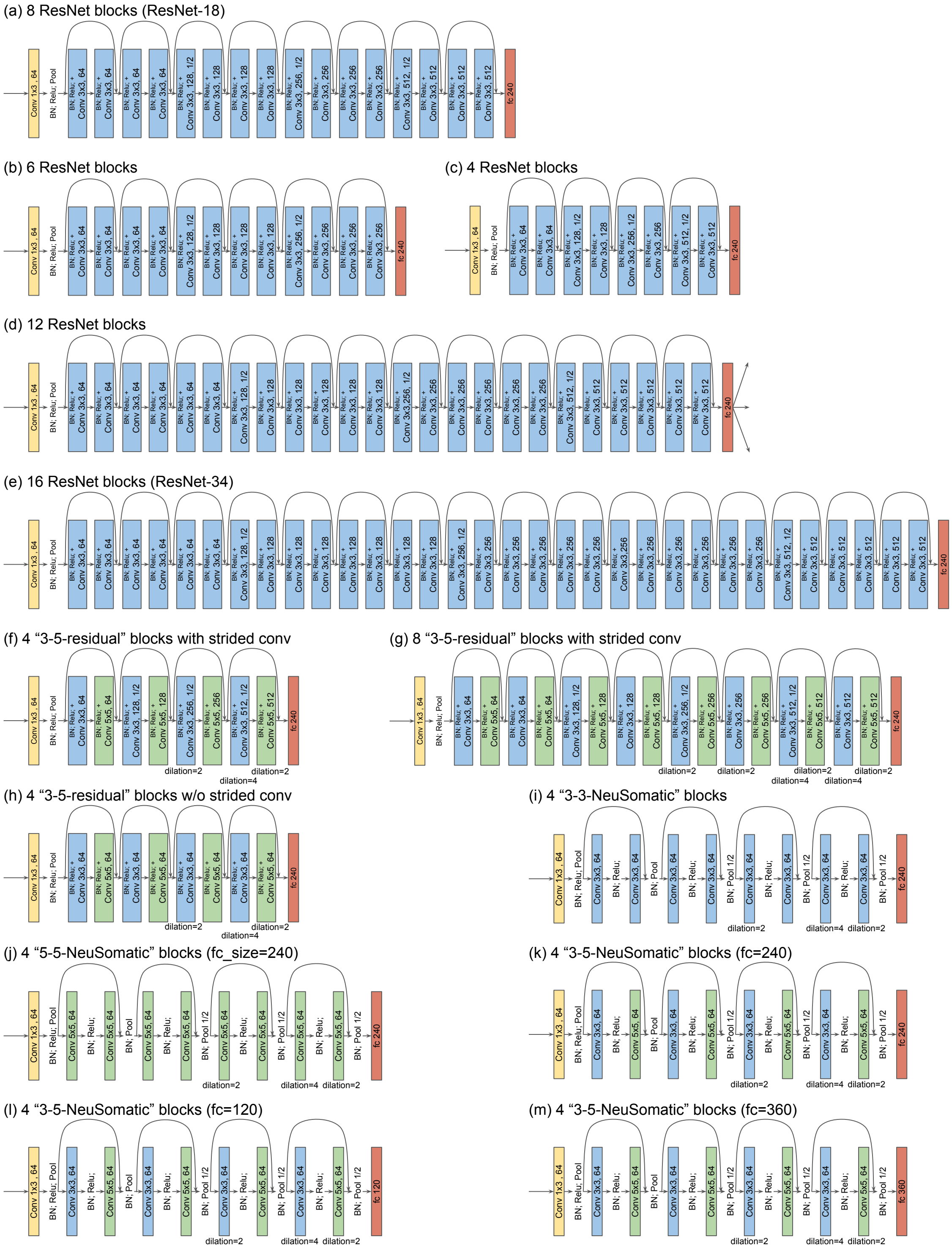

Supplementary Figure 13: Different network architectures tested. (a-e) ResNet architectures with different number of pre-activation residual blocks with default 3×3 conv layers. Here, strided convolutions are used with channel expansions. (f, g) Multiple customized residual blocks with 3×3 and 5×5 conv layers and some dilated convultions. Here, strided convolutions are used with channel expansions. (h) Four customized residual blocks with 3×3 and 5×5 conv layers and some dilated convultions. Here, no strided convolutions are used. (i-m) NeuSomatic residual architecture with different residual blocks and fully-connected sizes.

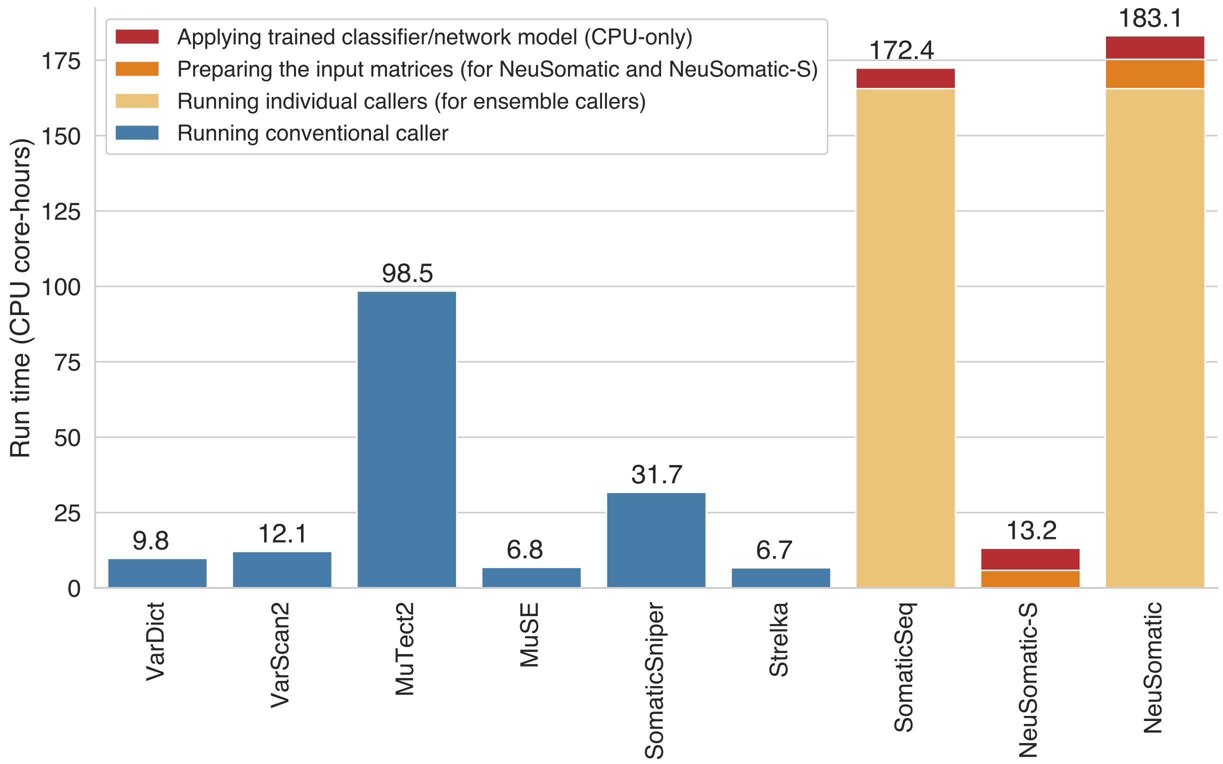

Supplementary Figure 14: Run-time comparison of different somatic mutation detection algorithms. CPU core-hours are shown for predicting somatic mutations on a 125× whole-exome sequencing dataset.

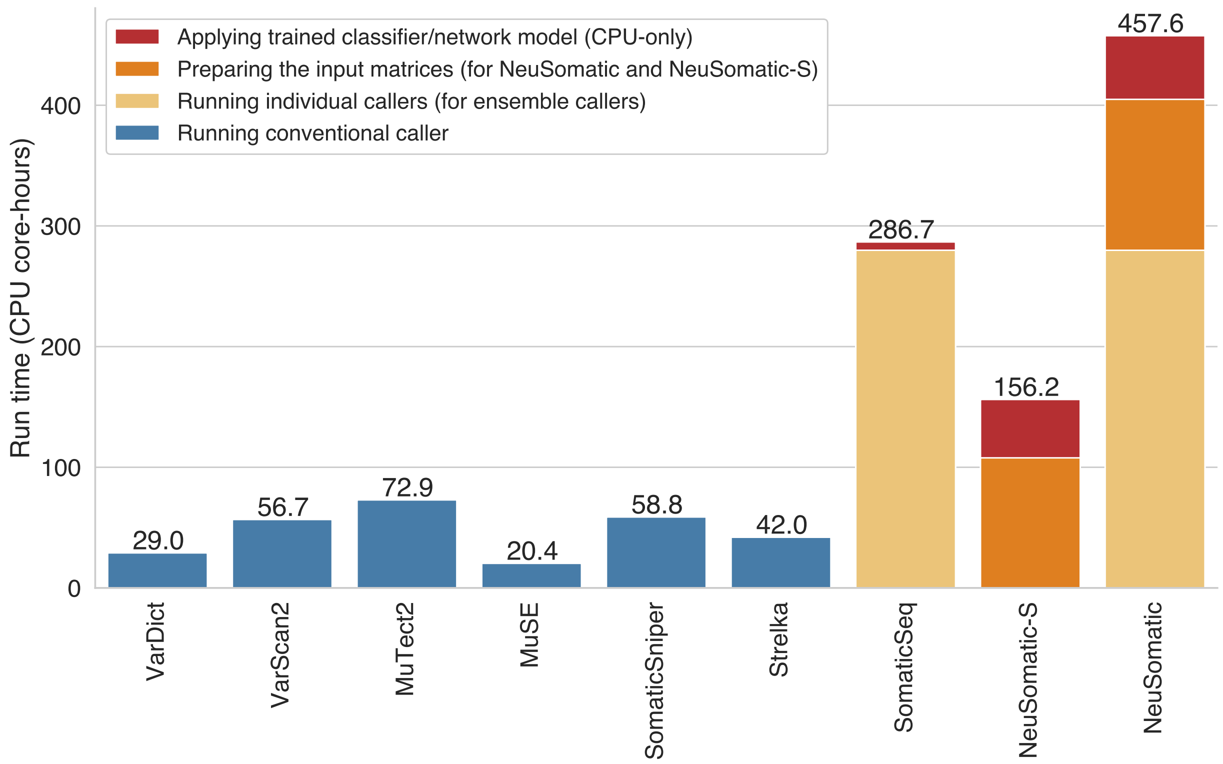

Supplementary Figure 15: Run-time comparison of different somatic mutation detection algorithms. CPU core-hours are shown for predicting somatic mutations on a 30× whole-genome sequencing dataset.

Supplementary Tables

Supplementary Table 1: Performance of different somatic mutation detection methods on Platinum two sample mix dataset. For each method we report the precision, recall and F1-score for the quality score threshold in precision-recall curve which achieves highest F1. (RC: Recall, PR: Precision, F1: F1-score)

**Method**	**50% Tumor 100% Normal**			**70% Tumor 95% Normal**			**50% Tumor 95% Normal**			**25% Tumor 95% Normal**		
	RC (%)	PR (%)	F1 (%)	RC (%)	PR (%)	F1 (%)	RC (%)	PR (%)	F1 (%)	RC (%)	PR (%)	F1 (%)
	**SNV**											
VarDict	97.9	92.1	94.9	72.5	93.0	81.5	72.3	92.5	81.2	68.9	91.8	78.7
VarScan2	99.1	92.2	95.5	96.2	92.9	94.5	94.3	93.3	93.8	52.9	94.0	67.7
MuTect2	89.9	94.0	91.9	22.7	92.8	36.4	22.6	93.1	36.4	19.7	93.8	32.5
MuSE	97.8	92.7	95.2	47.8	94.3	63.5	41.0	94.3	57.2	19.9	93.9	32.9
SomaticSniper	95.8	**100**	97.9	90.1	87.0	88.5	86.7	87.2	87.0	38.3	79.9	51.8
Strelka	99.2	92.3	95.6	98.7	93.2	95.9	98.9	92.8	95.7	**97.0**	90.4	93.6
SomaticSeq	98.1	97.0	97.5	95.3	96.6	95.9	94.7	96.8	95.7	83.6	96.7	89.7
NeuSomatic-S	99.3	99.4	99.4	99.5	99.4	99.5	99.3	99.4	99.3	96.9	98.8	97.9
NeuSomatic	**99.5**	99.5	**99.5**	**99.6**	**99.5**	**99.6**	**99.5**	**99.5**	**99.5**	**97.0**	**99.0**	**98.0**
	**INDEL**											
VarDict	74.0	95.8	83.5	57.2	95.1	71.4	54.6	94.8	69.3	49.3	89.2	63.5
VarScan2	85.0	98.3	91.1	86.0	98.1	91.6	77.0	98.4	86.4	33.7	**98.5**	50.2
MuTect2	66.5	97.8	79.2	25.7	97.2	40.6	24.8	97.5	39.5	17.3	97.9	29.5
Strelka	92.5	96.8	94.6	93.4	96.9	95.1	91.1	96.9	93.9	73.4	96.7	83.5
SomaticSeq	89.9	**99.3**	94.3	88.1	**99.3**	93.4	82.4	**99.3**	90.1	61.0	**98.5**	75.3
NeuSomatic-S	95.7	97.0	96.3	96.5	97.3	96.9	95.5	96.7	96.1	86.9	93.5	90.1
NeuSomatic	**95.8**	97.4	**96.6**	**96.9**	97.5	**97.2**	**95.7**	96.8	**96.3**	**87.7**	93.9	**90.7**

Supplementary Table 2: Performance of different somatic mutation detection methods on Dream Challenge Stage 3 dataset. For each method we report the precision, recall and F1-score for the quality score threshold in precision-recall curve which achieves highest F1. (RC: Recall, PR: Precision, F1: F1-score)

**Method**	**100% Tumor 100% Normal**			**50% Tumor 100% Normal**			**70% Tumor 95% Normal**			**50% Tumor 95% Normal**			**25% Tumor 95% Normal**		
	RC (%)	PR (%)	F1 (%)	RC (%)	PR (%)	F1 (%)	RC (%)	PR (%)	F1 (%)	RC (%)	PR (%)	F1 (%)	RC (%)	PR (%)	F1 (%)
	**SNV**														
VarDict	81.9	85.8	83.8	71.7	77.5	74.5	69.9	79.4	74.4	61.9	76.0	68.3	35.9	64.2	46.1
VarScan2	79.8	87.4	83.4	61.1	84.6	71.0	64.4	87.3	74.1	46.3	84.6	59.9	13.5	53.1	21.6
MuTect2	86.4	97.0	91.4	69.2	98.3	81.2	47.5	96.2	63.6	37.7	**97.7**	54.4	14.7	**99.7**	25.6
MuSE	89.8	97.0	93.3	72.1	94.4	81.8	59.4	91.2	71.9	49.1	86.3	62.6	20.9	91.2	34.0
SomaticSniper	75.7	**100**	86.2	30.3	**100**	46.5	48.6	93.4	63.9	27.5	94.8	42.7	2.2	94.6	4.3
Strelka	89.9	94.1	91.9	69.2	91.9	79.0	77.4	95.6	85.5	66.6	91.8	77.2	38.7	82.1	52.6
SomaticSeq	93.5	98.3	95.9	78.7	97.6	87.1	86.3	97.1	91.4	74.5	**97.1**	84.3	40.8	94.4	56.9
NeuSomatic-S	91.5	97.4	94.4	75.7	92.9	83.4	83.0	95.9	89.0	73.5	93.3	82.2	47.9	82.4	60.6
NeuSomatic	**94.0**	98.5	**96.2**	**79.5**	96.9	**87.3**	**87.1**	97.0	**91.8**	**77.3**	95.7	**85.5**	**48.5**	87.0	**62.3**
	**INDEL**														
VarDict	75.7	46.2	57.4	68.2	44.2	53.6	68.9	43.2	53.1	60.7	42.9	50.3	33.6	38.6	35.9
VarScan2	55.7	65.8	60.3	36.6	63.8	46.5	41.2	65.7	50.6	27.1	64.1	38.1	6.5	36.5	11.0
MuTect2	83.2	92.9	87.8	68.7	91.6	78.5	44.1	93.6	60.0	37.2	90.6	52.8	14.8	**94.3**	25.6
Strelka	68.6	88.5	77.3	41.5	88.1	56.5	52.1	85.0	64.6	39.0	79.2	52.2	13.6	73.9	22.9
SomaticSeq	90.2	**95.0**	92.5	74.3	**94.5**	83.2	80.4	**94.7**	87.0	69.6	**93.6**	79.8	35.5	93.8	51.5
NeuSomatic-S	84.5	90.2	87.2	72.5	89.5	80.1	78.9	88.5	83.4	71.4	88.9	79.2	54.9	76.7	64.0
NeuSomatic	**90.7**	96.4	**93.5**	**81.2**	90.3	**85.5**	85.2	**93.5**	89.2	**79.4**	**90.1**	**84.4**	**57.3**	81.6	**67.3**

Supplementary Table 3: Performance of different somatic mutation detection methods on Dream Challenge Stage 4 dataset. For each method we report the precision, recall and F1 score for the quality score threshold in precision-recall curve which achieves highest F1. (RC: Recall, PR: Precision, F1: F1-score)

**Method**	**100% Tumor 100% Normal**			**50% Tumor 100% Normal**			**70% Tumor 95% Normal**			**50% Tumor 95% Normal**			**25% Tumor 95% Normal**		
	RC (%)	PR (%)	F1 (%)	RC (%)	PR (%)	F1 (%)	RC (%)	PR (%)	F1 (%)	RC (%)	PR (%)	F1 (%)	RC (%)	PR (%)	F1 (%)
	**SNV**														
VarDict	76.7	78.5	77.6	43.7	80.1	56.6	57.0	74.1	64.4	39.6	71.6	51.0	12.9	61.8	21.4
VarScan2	64.1	78.1	70.4	32.3	69.3	44.1	38.9	73.7	50.9	21.4	68.0	32.5	5.3	3.1	3.9
MuTect2	68.5	97.2	80.3	33.5	98.2	50.0	36.3	**97.2**	52.8	21.8	**97.8**	35.6	4.8	**98.7**	9.1
MuSE	78.0	77.8	77.9	43.4	81.0	56.5	42.2	79.0	55.0	28.7	77.9	42.0	8.5	83.0	15.4
SomaticSniper	46.7	**100**	63.7	9.0	**100**	16.5	22.8	89.4	36.3	7.7	86.8	14.1	0.4	83.3	0.8
Strelka	68.8	86.9	76.8	35.0	78.4	48.4	50.2	77.6	60.9	33.0	72.5	45.4	14.4	18.2	16.1
SomaticSeq	85.1	95.9	90.2	52.0	93.0	66.7	66.7	93.4	77.8	48.1	93.3	63.5	16.9	86.2	28.3
NeuSomatic-S	80.7	92.7	86.3	45.2	83.2	58.6	61.5	89.1	72.8	45.0	82.9	58.3	19.5	53.2	28.6
NeuSomatic	**86.7**	95.9	**91.1**	**52.3**	92.4	**66.8**	**68.9**	94.0	**79.5**	**51.6**	90.3	**65.7**	**22.5**	71.4	**34.2**
	**INDEL**														
VarDict	74.9	52.6	61.8	43.9	52.5	47.8	56.9	50.3	53.4	40.3	49.0	44.2	13.2	36.9	19.4
VarScan2	50.4	55.9	53.0	19.7	53.7	28.8	27.8	49.9	35.7	14.1	41.2	21.0	3.2	12.9	5.1
MuTect2	69.5	94.7	80.1	36.1	98.1	52.8	37.1	97.1	53.7	24.1	**98.0**	38.7	5.8	**98.2**	11.0
Strelka	59.2	79.2	67.8	25.6	81.1	38.9	38.6	72.6	50.4	24.2	62.3	34.9	6.3	9.1	7.4
SomaticSeq	85.0	**98.6**	91.3	48.1	**98.6**	64.6	63.3	**98.6**	77.1	44.8	97.9	61.5	14.6	94.8	25.3
NeuSomatic-S	83.1	92.1	87.4	66.7	89.4	76.4	74.7	90.1	81.7	67.3	85.9	75.5	46.9	78.0	58.6
NeuSomatic	**89.8**	95.7	**92.6**	**71.6**	89.4	**79.5**	**78.8**	93.4	**85.5**	**71.4**	87.5	**78.6**	**48.0**	79.4	**59.9**

Supplementary Table 4: Performance of different somatic mutation detection methods on Platinum tumor spike dataset. For each method we report the precision, recall and F1-score for the quality score threshold in precision-recall curve which achieves highest F1. (RC: Recall, PR: Precision, F1: F1-score)

	**SNV**			**INDEL**		
	RC (%)	PR (%)	F1 (%)	RC (%)	PR (%)	F1 (%)
VarDict	58.6	96.8	73.0	40.1	81.2	53.6
VarScan2	38.6	99.1	55.6	25.4	94.7	40.1
MuTect2	54.3	**99.8**	70.3	32.7	**99.4**	49.2
MuSE	34.3	**99.8**	51.1	-	-	-
SomaticSniper	17.6	97.7	29.8	-	-	-
Strelka	65.0	94.0	76.9	39.4	89.8	54.8
SomaticSeq	60.3	99.7	75.1	46.4	96.3	62.6
NeuSomatic-S	**71.9**	92.5	**80.9**	**55.6**	81.1	66.0
NeuSomatic	71.6	92.9	**80.9**	**55.6**	83.5	**66.7**

Supplementary Table 5: Performance of different somatic mutation detection methods on whole-exome sample mix dataset. For each method we report the precision, recall and F1-score for the quality score threshold in precision-recall curve which achieves highest F1. (RC: Recall, PR: Precision, F1: F1-score)

**Method**	**50% Tumor 100% Normal**			**70% Tumor 95% Normal**			**50% Tumor 95% Normal**			**25% Tumor 95% Normal**		
	RC (%)	PR (%)	F1 (%)	RC (%)	PR (%)	F1 (%)	RC (%)	PR (%)	F1 (%)	RC (%)	PR (%)	F1 (%)
	**SNV**											
VarDict	95.5	99.1	97.3	57.8	98.7	72.9	56.8	98.7	72.1	54.1	98.5	69.9
VarScan2	98.0	99.3	98.7	85.4	99.3	91.9	84.0	99.3	91.1	69.0	99.4	81.5
MuTect2	90.2	99.3	94.6	7.0	97.7	13.1	7.0	97.9	13.1	6.4	98.5	12.1
MuSE	2.2	**100**	4.4	0.1	87.5	0.2	0.2	**100**	0.3	0.1	**100**	0.2
SomaticSniper	95.1	**100**	97.5	77.9	**100**	87.6	76.1	**100**	86.4	45.8	**100**	62.9
Strelka	97.6	99.1	98.4	95.5	97.8	96.6	93.6	96.6	95.1	64.1	93.1	75.9
SomaticSeq	98.4	99.0	98.7	87.5	98.7	92.8	86.9	98.8	92.4	80.6	98.4	88.6
NeuSomatic-S	98.6	99.5	99.1	98.6	99.5	99.0	**98.6**	99.3	**98.9**	**95.9**	98.1	**97.0**
NeuSomatic	**98.9**	99.6	**99.3**	**98.7**	99.5	**99.1**	98.3	99.5	**98.9**	95.7	97.8	96.7
	**INDEL**											
VarDict	74.0	96.3	83.7	43.1	90.2	58.3	41.2	94.1	57.3	36.3	82.4	50.4
VarScan2	78.0	**99.4**	87.4	68.2	**99.0**	80.8	65.2	**98.6**	78.5	48.1	**99.0**	64.8
MuTect2	58.1	98.0	72.9	7.5	80.0	13.7	7.5	69.6	13.5	5.6	82.8	10.5
Strelka	80.8	98.3	**88.7**	73.1	98.7	84.0	73.8	90.8	81.4	61.2	82.1	70.1
SomaticSeq	79.7	94.5	86.4	70.3	94.7	80.7	70.6	94.4	80.7	58.2	94.0	71.9
NeuSomatic-S	83.9	90.7	87.1	82.5	93.1	87.5	79.4	93.2	85.8	69.2	91.9	78.9
NeuSomatic	**86.2**	91.1	88.6	**83.6**	93.7	**88.4**	**84.8**	92.1	**88.3**	**72.4**	89.6	**80.1**

Supplementary Table 6: Performance of different somatic mutation detection methods on targeted panel dataset. For each method we report the precision, recall and F1-score for the quality score threshold in precision-recall curve which achieves highest F1. (RC: Recall, PR: Precision, F1: F1-score)

**Method**	**50% Tumor 100% Normal**			**70% Tumor 95% Normal**			**50% Tumor 95% Normal**			**25% Tumor 95% Normal**		
	RC (%)	PR (%)	F1 (%)	RC (%)	PR (%)	F1 (%)	RC (%)	PR (%)	F1 (%)	RC (%)	PR (%)	F1 (%)
	**SNV**											
VarDict	98.4	98.4	98.4	63.9	98.7	77.6	63.9	98.7	77.6	59.8	97.3	74.1
VarScan2	98.4	98.4	98.4	91.8	98.2	94.9	91.8	98.2	94.9	84.4	99.0	91.2
MuTect2	94.3	98.3	96.2	10.7	**100**	19.3	10.7	**100**	19.3	10.7	**100**	19.3
MuSE	1.6	**100**	3.2	-	-	-	-	-	-	-	-	-
SomaticSniper	97.5	**100**	98.8	87.7	**100**	93.4	86.9	**100**	93.0	63.1	**100**	77.4
Strelka	98.4	98.4	98.4	97.5	98.3	97.9	97.5	95.2	96.4	69.7	94.4	80.2
SomaticSeq	97.5	98.3	97.9	91.8	98.2	94.9	91.8	98.2	94.9	91.8	98.2	94.9
NeuSomatic-S	**99.2**	**100**	**99.6**	**99.2**	**100**	**99.6**	**99.2**	**100**	**99.6**	**98.4**	99.2	98.8
NeuSomatic	**99.2**	99.2	99.2	**99.2**	99.2	99.2	**99.2**	99.2	99.2	**98.4**	**100**	**99.2**

Supplementary Table 7: Performance of different somatic mutation detection methods on PacBio dataset. For each method we report the precision, recall and F1-score for the quality score threshold in precision-recall curve which achieves highest F1. (RC: Recall, PR: Precision, F1: F1-score)

**Method**	**50% Tumor Purity 95% Normal Purity**						**30% Tumor Purity 95% Normal Purity**						**20% Tumor Purity 95% Normal Purity**					
	**SNV**			**INDEL**			**SNV**			**INDEL**			**SNV**			**INDEL**		
	RC (%)	PR (%)	F1 (%)	RC (%)	PR (%)	F1 (%)	RC (%)	PR (%)	F1 (%)	RC (%)	PR (%)	F1 (%)	RC (%)	PR (%)	F1 (%)	RC (%)	PR (%)	F1 (%)
VarDict	57.7	94.3	71.6	23.3	49.5	31.7	49.4	82.5	61.8	10.2	10.0	10.1	45.7	55.0	49.9	7.0	0.7	1.3
NeuSomatic-S	**97.6**	**98.6**	**98.1**	**83.8**	**88.7**	**86.2**	**93.4**	**98.7**	**95.9**	**58.1**	**69.6**	**63.3**	**74.6**	**97.5**	**84.5**	**26.6**	**42.7**	**32.7**

Supplementary Table 8: Performance of different somatic mutation detection methods on real dataset COLO-829.

**Method**	**Number of Calls**	**Recall**	**Extrapolated Precision**	**Extrapolated F1**
VarDict	102712	94.1	37.6	53.7
VarScan2	62824	98.9	72.1	83.4
MuTect2	40405	96.9	94.7	95.8
MuSE	45857	**99.8**	92.8	96.2
SomaticSniper	46500	99.3	90.5	94.7
Strelka2	42818	99.1	94.9	97.0
SomaticSeq	39431	98.9	99.1	99.4
NeuSomatic-S	35413	89.0	88.3	88.7
NeuSomatic	**37843**	99.6	**99.9**	**99.7**

Supplementary Table 9: Performance of different somatic mutation detection methods on real dataset CLL1.

**Method**	**Number of Calls**	**Recall**	**Extrapolated Precision**	**Extrapolated F1**
VarDict	33418	88.0	17.8	29.6
VarScan2	11781	**90.4**	52.2	66.2
MuTect2	4382	82.4	73.4	77.6
MuSE	7500	89.6	67.6	77.0
SomaticSniper	8451	89.6	63.0	74.0
Strelka2	3575	90.2	86.6	88.4
SomaticSeq	3579	87.8	81.7	84.7
NeuSomatic-S	3224	88.4	81.8	84.9
NeuSomatic	**2581**	89.0	**97.9**	**93.2**

Supplementary Table 10: Performance of different somatic mutation detection methods on real dataset TCGA-AZ-6601.

**Method**	**Number of Calls**	**Recall**	**Extrapolated Precision**	**Extrapolated F1**
VarDict	3747	74.6	62.8	68.2
VarScan2	5041	98.2	63.9	77.5
MuTect2	3547	99.7	97.9	98.8
MuSE	3433	99.6	98.8	99.2
SomaticSniper	**1878**	65.7	98.7	78.8
Strelka2	3799	**100**	93.7	96.8
SomaticSeq	5275	**100**	71.7	83.5
NeuSomatic-S	3636	99.7	86.0	92.3
NeuSomatic	3401	99.9	**99.3**	**99.6**

Supplementary Table 11: List of 261 TCGA cancer samples used for Microsoft Azure experiment. Samples are taken across three cancer types: colorectal adenocarcinoma (COAD), ovarian serus adenocarcinoma (OV), and cervical squamous cell carcinoma and endocervical adenocarcinoma (CESC).

**Cancer Type**	**Sample IDs**				
COAD	TCGA-A6-2672	TCGA-A6-6653	TCGA-AA-3492	TCGA-AA-3510	TCGA-AA-3663
	TCGA-AA-3713	TCGA-AD-5900	TCGA-AD-6889	TCGA-AD-6895	TCGA-AD-6964
	TCGA-AU-6004	TCGA-AY-6197	TCGA-AZ-4315	TCGA-AZ-6598	TCGA-AZ-6599
	TCGA-AZ-6601	TCGA-CA-6716	TCGA-CA-6717	TCGA-CA-6718	TCGA-CK-4950
	TCGA-CK-4952	TCGA-CK-5913	TCGA-CK-5916	TCGA-CM-4743	TCGA-CM-4746
	TCGA-CM-5861	TCGA-CM-6162	TCGA-CM-6674	TCGA-CM-6678	TCGA-D5-6540
	TCGA-D5-6927	TCGA-D5-6928	TCGA-D5-6930	TCGA-D5-6931	TCGA-DM-A0XD
	TCGA-DM-A1D4	TCGA-DM-A1DA	TCGA-F4-6570	TCGA-F4-6703	TCGA-F4-6856
	TCGA-G4-6299	TCGA-G4-6304	TCGA-G4-6309	TCGA-G4-6586	TCGA-G4-6588
	TCGA-G4-6628				
CESC	TCGA-C5-A2LS	TCGA-C5-A2LX	TCGA-C5-A2M1	TCGA-C5-A2M2	TCGA-C5-A3HF
	TCGA-C5-A7CG	TCGA-C5-A7CH	TCGA-C5-A7CJ	TCGA-C5-A7CK	TCGA-C5-A7CL
	TCGA-C5-A7CM	TCGA-C5-A7CO	TCGA-C5-A7UC	TCGA-C5-A7UE	TCGA-C5-A7UH
	TCGA-C5-A7X3	TCGA-DG-A2KH	TCGA-DG-A2KK	TCGA-DG-A2KL	TCGA-DG-A2KM
	TCGA-DS-A3LQ	TCGA-DS-A5RQ	TCGA-DS-A7WF	TCGA-DS-A7WH	TCGA-DS-A7WI
	TCGA-EA-A1QS	TCGA-EA-A3HQ	TCGA-EA-A3HR	TCGA-EA-A3HT	TCGA-EA-A3HU
	TCGA-EA-A3QD	TCGA-EA-A3QE	TCGA-EA-A3Y4	TCGA-EA-A410	TCGA-EA-A411
	TCGA-EA-A439	TCGA-EA-A43B	TCGA-EA-A5FO	TCGA-EA-A5O9	TCGA-EA-A5ZD
	TCGA-EA-A5ZE	TCGA-EA-A5ZF	TCGA-EA-A6QX	TCGA-EA-A78R	TCGA-EK-A2GZ
	TCGA-EK-A2H0	TCGA-EK-A2H1	TCGA-EK-A2IP	TCGA-EK-A2PG	TCGA-EK-A2PI
	TCGA-EK-A2PL	TCGA-EK-A2PM	TCGA-EK-A2R7	TCGA-EK-A2R8	TCGA-EK-A2R9
	TCGA-EK-A2RA	TCGA-EK-A2RB	TCGA-EK-A2RC	TCGA-EK-A2RD	TCGA-EK-A2RE
	TCGA-EK-A2RJ	TCGA-EK-A2RK	TCGA-EK-A2RL	TCGA-EK-A2RN	TCGA-EK-A2RO
	TCGA-EK-A3GJ	TCGA-EK-A3GK	TCGA-EK-A3GM	TCGA-EK-A3GN	TCGA-EX-A1H6
	TCGA-EX-A3L1	TCGA-EX-A69L	TCGA-EX-A69M	TCGA-FU-A2QG	TCGA-FU-A3EO
	TCGA-FU-A3HY	TCGA-FU-A3NI	TCGA-FU-A3TQ	TCGA-FU-A3TX	TCGA-FU-A3WB
	TCGA-FU-A3YQ	TCGA-FU-A40J	TCGA-FU-A5XV	TCGA-FU-A770	TCGA-HG-A2PA
	TCGA-HM-A3JK	TCGA-IR-A3L7	TCGA-IR-A3LA	TCGA-IR-A3LB	TCGA-IR-A3LC
	TCGA-IR-A3LF	TCGA-IR-A3LH	TCGA-IR-A3LI	TCGA-IR-A3LK	TCGA-IR-A3LL
	TCGA-JW-A5VG	TCGA-JW-A5VH	TCGA-JW-A5VI	TCGA-JW-A5VJ	TCGA-JW-A5VK
	TCGA-JW-A5VL	TCGA-JW-A69B	TCGA-JW-A852	TCGA-JX-A3PZ	TCGA-JX-A3Q0
	TCGA-JX-A3Q8	TCGA-JX-A5QV	TCGA-LP-A4AU	TCGA-LP-A4AV	TCGA-LP-A4AW
	TCGA-LP-A4AX	TCGA-LP-A5U2	TCGA-LP-A5U3	TCGA-LP-A7HU	TCGA-MU-A5YI
	TCGA-Q1-A5R1	TCGA-Q1-A5R2	TCGA-Q1-A5R3	TCGA-Q1-A6DT	TCGA-Q1-A6DV
	TCGA-Q1-A6DW	TCGA-Q1-A73O	TCGA-Q1-A73P	TCGA-Q1-A73Q	TCGA-Q1-A73R
	TCGA-Q1-A73S	TCGA-R2-A69V	TCGA-RA-A741	TCGA-UC-A7PD	TCGA-UC-A7PF
	TCGA-WL-A834				
OV	TCGA-04-1332	TCGA-04-1336	TCGA-04-1343	TCGA-04-1346	TCGA-04-1347
	TCGA-04-1348	TCGA-04-1349	TCGA-04-1361	TCGA-04-1362	TCGA-04-1542
	TCGA-09-0366	TCGA-09-0369	TCGA-10-0930	TCGA-10-0933	TCGA-10-0935
	TCGA-13-0723	TCGA-13-0724	TCGA-13-0726	TCGA-13-0755	TCGA-13-0760
	TCGA-13-0765	TCGA-13-0791	TCGA-13-0795	TCGA-13-0800	TCGA-13-0804
	TCGA-13-0807	TCGA-13-0884	TCGA-13-0885	TCGA-13-0887	TCGA-13-0890
	TCGA-13-0893	TCGA-13-0894	TCGA-13-0897	TCGA-13-0903	TCGA-13-0910
	TCGA-13-0912	TCGA-13-0920	TCGA-13-0924	TCGA-13-1403	TCGA-13-1404
	TCGA-13-1405	TCGA-13-1411	TCGA-13-1412	TCGA-13-1481	TCGA-13-1482
	TCGA-13-1483	TCGA-13-1488	TCGA-13-1489	TCGA-13-1491	TCGA-13-1497
	TCGA-13-1498	TCGA-13-1499	TCGA-13-1506	TCGA-13-1507	TCGA-13-1509
	TCGA-23-1021	TCGA-23-1022	TCGA-23-1117	TCGA-23-1118	TCGA-23-1123
	TCGA-23-1124	TCGA-24-0966	TCGA-24-0980	TCGA-24-1103	TCGA-24-1104
	TCGA-24-1413	TCGA-24-1416	TCGA-24-1417	TCGA-24-1418	TCGA-24-1424
	TCGA-24-1425	TCGA-24-1426	TCGA-24-1427	TCGA-24-1428	TCGA-24-1435
	TCGA-24-1436	TCGA-24-1463	TCGA-24-1464	TCGA-24-1469	TCGA-24-1470
	TCGA-24-1562	TCGA-24-1616	TCGA-25-1315	TCGA-25-1316	

Supplementary Table 12: Performance analysis of different network architectures shown in Supplementary Figure 13. Here, all the networks are assessed with batch size of 1000 and after 600 epochs training.

**id**	**Network architecture**	**Network parameters**	**Seconds per epoch of 1M candidates**	**GPU Memory** (GB)	**SNV F1-score (%)**	**INDEL F1-score (%)**
a	8 ResNet blocks (ResNet-18)	13.6M	446	5	89.65	86.87
b	6 ResNet blocks	5.2M	157	3.7	89.28	86.50
c	4 ResNet blocks	7.4M	209	3.5	89.18	**87.15**
d	12 ResNet blocks	19.9M	686	5.2	89.43	86.34
e	16 ResNet blocks (ResNet-34)	23.8M	853	7	87.99	84.87
f	4 "3-5-residual" blocks with strided conv	12.9M	366	9.4	89.82	86.87
g	8 "3-5-residual" blocks with strided conv	24.7M	418	9.4	**89.88**	**87.15**
h	4 "3-5-residual" blocks w/o strided conv	0.9M	70	7.2	88.30	86.44
i	4 "3-3-NeuSomatic" blocks	**0.6M**	**45**	**2.4**	89.43	86.80
j	4 "5-5-NeuSomatic" blocks	1.1M	176	9.2	89.59	86.85
k	4 "3-5-NeuSomatic" blocks (fc=240)	0.9M	117	9.3	89.64	86.92
l	4 "3-5-NeuSomatic" blocks (fc=120)	0.7M	115	9.3	89.23	86.44
m	4 "3-5-NeuSomatic" blocks (fc=360)	1.0M	115	9.3	89.30	86.90
